# Sociotechnical Human Factors Involved in Remote Online Usability Testing of Two eHealth Interventions

**DOI:** 10.2196/humanfactors.4602

**Published:** 2016-02-03

**Authors:** Lori M Wozney, Pamela Baxter, Hilary Fast, Laura Cleghorn, Amos S Hundert, Amanda S Newton

**Affiliations:** ^1^ Centre for Research in Family Health IWK Health Centre Halifax, NS Canada; ^2^ School of Nursing Health Sciences Centre McMaster University Hamilton, ON Canada; ^3^ Edmonton Clinic Health Academy Department of Pediatrics, Faculty of Medicine & Dentistry University of Alberta Edmonton, AB Canada

**Keywords:** usability inspection, Web-conferencing, telemedicine, HCI design and evaluation methods, walk-through evaluation, real-time systems, human centered-computing

## Abstract

**Background:**

Research in the fields of human performance technology and human computer interaction are challenging the traditional macro focus of usability testing arguing for methods that help test moderators assess “use in context” (ie, cognitive skills, usability understood over time) and in authentic “real world” settings. Human factors in these complex test scenarios may impact on the quality of usability results being derived yet there is a lack of research detailing moderator experiences in these test environments. Most comparative research has focused on the impact of the physical environment on results, and rarely on how the sociotechnical elements of the test environment affect moderator and test user performance. Improving our understanding of moderator roles and experiences with conducting “real world” usability testing can lead to improved techniques and strategies

**Objective:**

To understand moderator experiences of using Web-conferencing software to conduct remote usability testing of 2 eHealth interventions.

**Methods:**

An exploratory case study approach was used to study 4 moderators’ experiences using Blackboard Collaborate for remote testing sessions of 2 different eHealth interventions. Data collection involved audio-recording iterative cycles of test sessions, collecting summary notes taken by moderators, and conducting 2 90-minute focus groups via teleconference. A direct content analysis with an inductive coding approach was used to explore personal accounts, assess the credibility of data interpretation, and generate consensus on the thematic structure of the results.

**Results:**

Following the convergence of data from the various sources, 3 major themes were identified: (1) moderators experienced and adapted to unpredictable changes in cognitive load during testing; (2) moderators experienced challenges in creating and sustaining social presence and untangling dialogue; and (3) moderators experienced diverse technical demands, but were able to collaboratively troubleshoot with test users.

**Conclusions:**

Results highlight important human-computer interactions and human factor qualities that impact usability testing processes. Moderators need an advanced skill and knowledge set to address the social interaction aspects of Web-based usability testing and technical aspects of conferencing software during test sessions. Findings from moderator-focused studies can inform the design of remote testing platforms and real-time usability evaluation processes that place less cognitive burden on moderators and test users.

##  Introduction

Traditional usability testing sessions for Internet-based (ie, eHealth) interventions focus on assessing the effectiveness, efficiency, and learnability of and user satisfaction with the intervention. These test situations typically involve in-person, lab-based, or field sessions [1-3]. While a lab setting allows for more experimental control and collection of various types of data during usability testing, it lacks the realism of a field setting. It also precludes deeper engagement from potential end users offered through field-based moderation. Usability moderators leverage many of the skills qualitative researchers already have (eg, building rapport, probing for clarity, getting below top-of-mind responses). To a large extent, successful usability testing depends on the skills of the person moderating the test, which Dumas and Loring suggest is “easy to do, hard to do well” [4].

Fieldwork that includes remote Web-based moderation is a novel approach to usability testing that could potentially mitigate some of the common problems experienced in lab-setting facilitation (eg, cost of maintaining a lab, less authentic or “real world” use contexts) [5]. However, any challenges experienced in computer-mediated communication between test users and moderator has a direct impact on the quality and accuracy of research findings and subsequent decisions about design. For testing of Internet-based interventions aimed at individuals with medically complex situations (eg, comorbidities, chronic illness, and/or frequent relapse cycles), the moderators’ ability to confidently use tools that help explore and communicate “use in context” experiences are critical to successful design [6].

Technologies that support real-time, remote collaboration have expanded usability testing possibilities to include geographically remote testing through Web-based moderation (eg, Morae and UserZoom remote usability testing platforms). During Web-based usability testing, the moderator and test user can be geographically separated but can still observe, prompt, and respond to questions in real-time. This approach may help to address the study of more complex eHealth interventions and difficulties that can arise from lab-based and other forms of field-testing when target users are: (1) needed from within a certain clinical population that is geographically dispersed; (2) have limitations in functioning and accessibility due to illness, often the reason for which the intervention was developed; and/or (3) are part of at-risk or age-sensitive groups (eg, minors who would be in school during typical “business hours”) that face challenges in travel, time, and cost of attending in-person lab testing [7]. Importantly, studies comparing lab-based testing with remote testing have consistently found no significant difference in usability performance results [8,9].

Web-based usability testing can involve synchronous (ie, moderators and test users are in same place [virtual or physical] at the same time), asynchronous (ie, automated, no real-time interaction), and blended (ie, asynchronous and synchronous) approaches. Remote, synchronous methods are proposed to be useful for usability testing early in the intervention development process. Real-time discussions between the moderator and user can be used to identify usability concerns while prototypes and user interface models are still under development [10] and may potentially save on development costs. While asynchronous, automated methods enable access to large data pools, the reliability of this testing approach has been questioned [11], and it is proposed that this approach may be more time-consuming for the novice tester and result in fewer usability problems being identified [8]. Automated testing methods alone are also not conducive to identifying what Andrezejczak [12] calls the “softer” subjective usability elements (eg, user preferences, misconceptions, underlying values, context variables, motivational attributes, affective attributes) that are better explored through synchronous inquiry methods with a moderator [13].

Web conferencing software packages (eg, GoToMeeting, Cisco WebEx, Microsoft NetMeeting or Lotus Sametime, Blackboard Collaborate, Adobe Connect Pro) are one option for remotely connecting with test users. Although the literature supporting Web-conferencing tools for online collaboration in higher education is extensive [14,15], published research on the use of these tools in moderating usability testing is limited. With the range of functionality provided by these software systems, there is potential to support a diverse range of remote usability session configurations and testing tasks. To date, published research has only begun to explore the role of social environment (ie, individuals present during testing) or the interactions between physical and social environments in usability testing. Study of physical usability test environments suggests that social context plays a substantial role in the quality of usability evaluation results [16,17]. Evaluator effect has been probed by van den Haak and de Jong [18] and interactions between test monitor and test users across multiple in-lab test scenarios were shown to have a significant effect on usability results. The detection of problems and selection of priority usability issues are subject to considerable individual variability [2]. These facets may be equally prominent during Web-based moderation, but there is little research exploring remote usability testing from the moderator’s point of view.

The purpose of this study was to: (1) understand moderator experiences using Web-conferencing software in the context of conducting remote usability testing; (2) compare and contrast moderator experiences using the same Web-conferencing software for 2 different Internet-based eHealth interventions; and (3) highlight important practical human-computer interactions qualities that may impact usability testing processes for other researchers.

## Methods

### Research Design and Usability Testing Context

**Figure 1:**
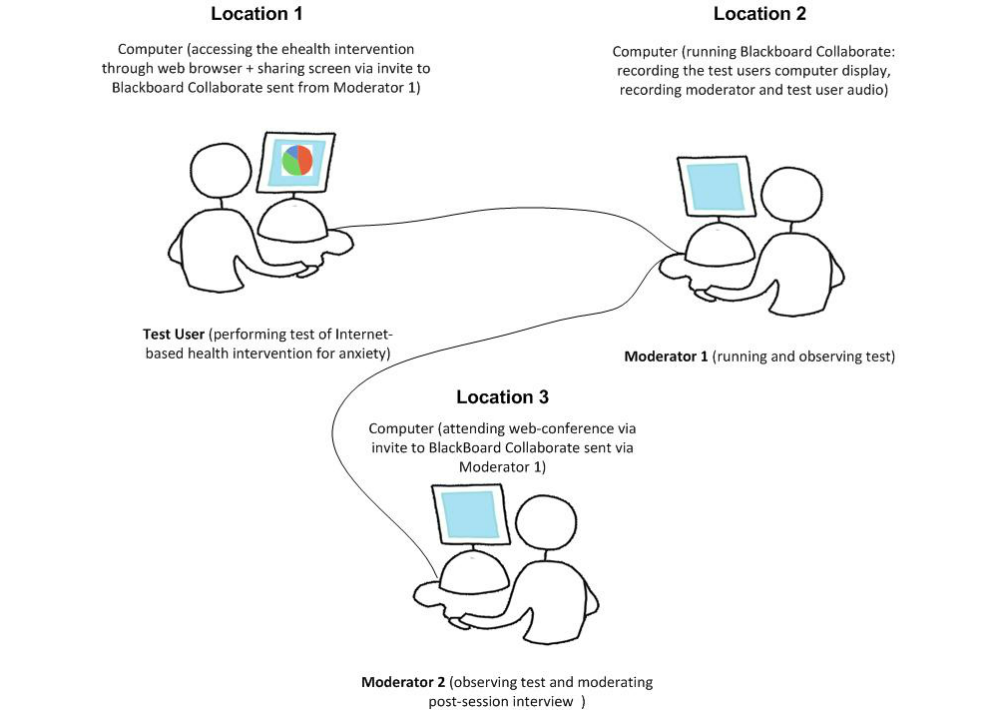
Web-conferencing test environment setup for ehealth Project 1. Moderator 1 controls recording and access privileges to test environment. Test User and Moderator 2, each in different geographic locations, act as “attendees” with different roles.

**Figure 2:**
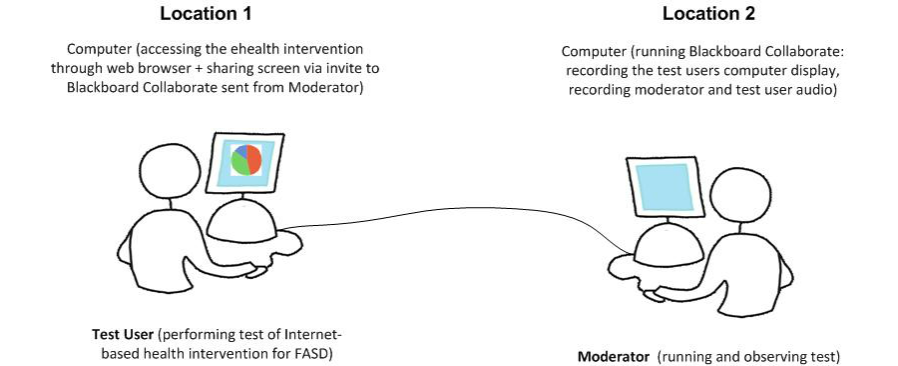
Web-conferencing test environment setup for ehealth Project 2. Moderator controls recording and access privileges. Test User “attends” the web-conference and shares screen so Moderator can observe actions.

A single case study approach was used to study the experiences of 4 moderators on 2 projects involving usability testing on eHealth interventions designed and delivered via a “smartsite” software platform called IRIS (intelligent research and intervention software) [19]. This approach allowed for rigorous exploration of the phenomena incorporating multiple perspectives and the dynamism of observations across time and projects [20,21]. Project 1 was an Internet-based anxiety treatment program for adolescents with anxiety disorders. Project 2 was an Internet-based intervention for caregivers of children with fetal alcohol spectrum disorders (FASD). Usability testing was conducted to improve the interventions in terms of the content (ie, therapeutic message, sequence of modules), aesthetics (ie, “look and feel,” appropriateness of images), and IRIS platform functionality (eg, customization abilities, site navigation tools, communication features). Usability testing protocols for both projects were approved by institutional research ethics boards and test users provided informed consent. A comparative summary of usability testing set up for the 2 projects is described in Table 1. Figures 1 and 2 illustrate the different usability test scenarios and roles for both projects.

**Table 1:** Comparison of usability project moderation.

Protocol Feature	Cycle	Project 1 (Anxiety)	Project 2 (FASD)
Number of cycles		2 (same group of test users for both cycles)	2 (new group of test users in each cycle)
Number of test users per cycle, n	Cycle 1	9 (4 youth, 5 clinicians)	10 (4 caregivers, 6 clinicians/health care professionals)
	Cycle 2	8 (4 youth, 4 clinicians)	8 (4 caregivers, 4 clinicians)
Dates of session	Cycle 1	June–July 2013	August–September 2013
	Cycle 2	September 2013	October–November 2013
Number of remote moderators in each session		2	1
Access to intervention prior to remote usability session		No	No
Software version		Blackboard Collaborate 9.7	Blackboard Collaborate 12.5
Average length of usability testing session		133 minutes^a^	62 minutes^b^
Location of moderator(s)		Ontario, Alberta	Nova Scotia
Estimated training time required for moderators to set up usability sessions		40 hours	20 hours
Location of test users		Nova Scotia, Alberta, British Columbia	British Columbia, New Brunswick, Alberta, Saskatchewan, Yukon, Ontario, Manitoba, Northwest Territories
Moderator(s) had prior experience as user in Web conferencing		Yes	Yes
Moderator(s) had prior experience moderating via Web conferencing		No	No
Moderator(s) had prior experience facilitating usability testing		No	No
Moderator(s) had prior experience in facilitating research interviews		Yes	Yes

^a^133 minutes=average time for Cycle 1 and Cycle 2 combined

^b^62 minutes=average time for Cycle 1 and Cycle 2 combined

During the usability testing, moderators used Blackboard Collaborate, a Web-conferencing system and one of the most advanced computer-mediated communication platforms on the market. The system was selected due to its low bandwidth, which accommodates slower user connection speeds making it more widely accessible for test users involved in the 2 projects. Interacting through the Blackboard Collaborate system is designed to mimic face-to-face contexts. Moderators and test users can share screens, indicate a desire to talk by clicking on a “raise hand” button, chat through instant messaging, and “draw” on the virtual whiteboard. The session moderator retains control of the various system tools, but he or she can share that control with others [22]. In addition to the functionality provided, Blackboard Collaborate was selected because it was an institutionally adopted tool at both main research institutions involved in the study, meaning no additional software licensing fees were required and there was technical support available on-site.

To prepare for using Blackboard Collaborate in the test sessions, moderators viewed demonstration videos, attended online tutorials, participated in mock sessions, and undertook several iterations of trial-and-error. To explore “use in context,” both projects configured test sessions to support blended usability testing techniques: “cognitive walk-through” (eg, user is given a task and the evaluator observes user’s intentions and the feedback provided by the system’s interface); “think-aloud” (ie, “novice” users verbalize their experiences as they work through tasks); and post-hoc interviews and self-report questionnaires.

### Data Collection

Prior to the first test session, session moderators sent an email to test users that provided technical instructions (eg, updating Java, testing audio) and study procedures. Test users in both projects were mailed USB headsets with noise-canceling microphones, if needed. Once logged into the session moderators had to walk users through “auto tuning” tests to ensure they were able to hear and be heard during the session. Test users “shared” their desktop so the moderator could observe them. Although the simultaneous camera feature was available, and offered to test users, none of the test users self-selected to employ this feature. Figure 1 shows an example of the moderator Web-conferencing environment for a Cycle 1 usability test session for Project 1. Project 1 involved 2 moderators in all but 1 test session. The first moderator focused on facilitating the main usability session walk-through tasks. The second moderator facilitated the post-session, open-ended question portion of the test session as more of an interviewer. A third moderator was present for 1 test session only as an observer to be able to provide feedback on usability test processes for the other moderators. In Project 2, only 1 moderator was present during all of the sessions. Moderators in both Projects 1 and 2 made detailed notes during each test session and created summary reports of key observations immediately following each test. Audio and/or video files for all sessions were recorded and saved as JAR files.

Focus groups via teleconference were held with all moderators at 2 points in time. The first was held 2 weeks after Cycle 2 usability test sessions for both projects were complete. Although an immediate debrief would have been ideal, coordinating a multi-site, multi-time-zone research team presented certain scheduling challenges. The second focus group was held 4 weeks later to allow time for incorporating feedback and review. Teleconferences were not digitally recorded, but detailed notes were taken by the first author. Notes included some verbatim statements and paraphrases of verbal statements. The first 90-minute focus group focused on a micro perspective of the data with each moderator describing their personal account and experiences. The over-arching exploratory question being: “What are moderators’ experiences using Web-conferencing for conducting remote usability testing?” An additional list of probative questions was circulated to all moderators 1 week prior to the initial focus group. The following list of questions was informally used as the focus group discussion guide:

1. How did Blackboard Collaborate support/hinder you as a researcher? Our team?
2. How do you think the tool supported/hindered our research participants?
3. Was there anything about the tool that surprised you? Really confused you?
4. Which testing activities (cognitive walk-through/think-aloud) was Blackboard Collaborate more useful for? Why?
5. How did it feel for you to be remote from the user/mediated by the computer?
6. Were you concerned about not having any visual cues, such as body language, to guide you? Why?
7. What (if any) ethical issues did you have with using this tool?
8. How do you think using this tool differed from what you would have done face to face?
9. Do you think you captured different types of data using this tool? If yes, in what way?
10. If another researcher was thinking about conducting remote usability sessions would you recommend this tool? Why? Why not?

The second 90-minute focus group occurred following the preliminary data analysis stage as part of planned member check-through debriefing and respondent validation as recommended by Koelsch [23]. During the second focus group, the first author guided the discussion toward theory development from a macro perspective (ie, exploring meaning of collective experiences). The discussion focused on: (1) assessing the credibility of preliminary data interpretation, (2) refining the proposed thematic structure, and (3) evaluating the suitability of examples appearing within the master list of themes [24]. Detailed notes were again taken. Member checks also occurred informally over several weeks during the normal course of observation and conversation with research team members over email, by phone, and in person.

## Results

We conducted an iterative thematic analysis whereby data were analyzed from all sources; the analysis was examined and reorganized, the reorganized data was synthesized, and the synthesis was then interpreted [25]. This inductive analytic approach strengthens the reliability of qualitative research [26]. In the first phase, features of each moderator’s experience were carefully detailed by a close reading of session transcripts, moderator session notes, and notes from the first focus group. A master list of emergent themes was drafted by the first author after the first focus group and circulated via email to all moderators to promote retrospection and exploration into any issues with the trustworthiness (ie, dependability, confirmability) of the synthesized master list. Written responses to this member check were returned by each moderator via email with suggested changes or clarifications integrated into the draft version. The second phase of interpretation involved exploring convergences and divergences within and between individual accounts. During the second focus group, points that were identified during member check that required clarification were discussed. This second iterative analysis phase allowed us to capture any interesting relationships, patterns, surprises, and inconsistencies among people and within and across sites [27]. Notes from the second focus group were used to further refine master theme examples and descriptions.

### Themes

Three major themes emerged from the converged data: (1) experiencing and adapting to unpredictable changes in cognitive load; (2) experiencing challenges in creating and sustaining social presence and untangling dialogue; and (3) collaboratively troubleshooting diverse technical needs and issues with test users. Moderators’ experiences were, overall, characterized by generally positive feelings and attitudes toward the experience of moderating usability testing remotely via Blackboard Collaborate. There was considerable congruence between themes, more so in some cases than others. The interdependence of themes makes it difficult to separate some examples into component parts. Test users shared similar experiences, and although there were idiosyncrasies that marked individual experience, differences between test users were generally characterized by the intensity or depth of these shared experiences.

#### Theme 1: Moderators Experienced and Adapted to Unpredictable Changes in Cognitive Load

Moderators all agreed that the range of communication features available in Blackboard Collaborate helped support interpretive aspects of usability testing. Test users not only identified problems and errors, but were also able to participate in impromptu interpretation of what problems meant or how they might be solved.

I was surprised at how often youth would stop and offer suggestions about how to improve things...they didn’t just point out problems...they had a lot of creative ideas about how to improve things...we could brainstorm together.

MOD 1

Often these spontaneous interpretive interactions between test users and moderators happened because moderators were able to “follow test users’ lead” by extemporaneously prompting for additional information or checking assumptions when indicated. Through the Blackboard Collaborate interface, moderators and test users could (via desktop computers) see, hear, show, capture, complete questionnaires, and engage in different kinds of interpretive dialogue. More importantly, the Blackboard Collaborate interface provided moderators with the opportunity to define when and how that interpretive dialogue took shape [28].

Conducting robust usability testing of a complex Internet-based intervention using Web-conferencing software that was new to test users did, however, create a cognitively demanding environment. Moderators had to manage concurrent use of Blackboard Collaborate plus the online intervention being tested, all while trying not to confuse learners or overload themselves. The real-time aspect meant that usability sessions were never predictable. The need for moderators and test users to divide their attention among auditory, textual, and visual material made high demands on limited working memory, creating at times a kind of “cognitive overload”:

Especially at the start of the session, when you were trying to get everything set up and working properly for their audio [and] *...*explain how the ‘think aloud’ process worked,*...*there was a lot to keep track of on the screen*...*One time a test user forgot to unmute and just started talking*...*we had no idea*...*There were quite a few interruptions in the first 5 to 10 minutes.” [MOD 1]

Moderators also had to adapt their approaches to each test user’s responses and needs. Moderators in Project 1, which involved the same group of test users in both testing cycles, noted that these challenges were greatly reduced during the second cycle of testing as everyone had more experience with Blackboard Collaborate and with the usability testing process. Moderators’ perceptions were that Cycle 2 was not effortless, but certainly more efficient:

[It was] more relaxed...conversation was more focused...more time could be spent exploring possible solutions to problems.... [We were] less anxious about technical problems...[and] didn’t feel as stressed.

MOD 2

One moderator noted how Blackboard Collaborate sessions meant “being ready for anything” and having to problem-solve “on the fly,” although moderators generally felt they were less affected by “cognitive overload.” All moderators talked about their use of the mute button that allowed them to listen without being heard. Moderators felt this “privacy” allowed them to keep the live testing space quiet and less distracting (eg, test users didn’t hear them drinking water, there was less background noise to distract the test user). All moderators provided anecdotal examples where test users themselves used the mute button to attend to something happening outside the test session (eg, receiving a phone call, checking on somebody in their house, eating lunch). Moderators in Project 1 used the “private chat” function as another channel to communicate between themselves without “disrupting” the test user:

Private chat was helpful...we could keep each other on track for time...When users were busy working on a task, we could private message each other. The test users didn’t see [that] we could check in with each other.

MOD 1

The ability to control and create these mini “offline” experiences in the online space meant moderators and test users could attend to other (and sometimes outside) impromptu demands. Moderators experienced fluctuating demands on their mental resources across test user sessions and testing cycles. Keeping test users on task, dealing with simultaneous tasks, and optimizing use of time during the session, required that moderators have considerable capacity to problem-solve in a complex collaborative environment.

#### Theme 2: Moderators Experienced Challenges in Creating and Sustaining a Sense of “Presence” and Untangling Dialogue

Moderators expressed different opinions about the quantity and nature of the social interaction, or sense of “presence,” that Blackboard Collaborate supported, both across the different projects and across iterative testing cycles. Table 2 outlines the main benefits and limitations experienced by moderators in creating and sustaining different facets of presence: social presence (ie, the sense of being with others), control (ie, the sense of interacting in an environment that is responsive to you), and personal presence (ie, the sense of immediacy or “being there”). Despite the synchronous nature of the exchanges in Blackboard Collaborate that mimic the interaction possibilities in face-to-face testing, there were still challenges for moderators and test users in terms of quickly establishing rapport in a virtual environment. The ability of test users to control the flow of communication to some degree (ie, choosing whether to use the camera tool, muting the session momentarily if needed, and completing the test from any location) meant that “presence” in the testing environment reflected test users’ own choices—not a predetermined “ideal” test environment.

**Table 2:** Examples of benefits and limitations of using Blackboard Collaborate.

Presence factors		Specific examples
Benefits	Anonymity	Moderators or test users might be more willing to share honestly or critically if less visible/identifiable.
	Test users have a sense of control	Moderators or test users could mute the session if they wanted to limit noise.
		Moderators could employ the “private chat” feature.
		Test users have control of when and where their session was held (some test users completed testing at home or at work).
	Authentic use in context	Test users could mute the session for reasons such as: check on children, take a phone call, speak to a coworker, eat.
		By having an unstandardized testing approach, the teams were given insights into the nature of technology use in people’s everyday lives and routines. This was valuable information about how the eHealth interventions being tested might also be used.
Limitations	Lack of visual cues, “personality,” or human element in the virtual space	The lack of visual cues led to moderators feeling they were checking in with the test user more than necessary. If there was silence, or no movement on the screen, moderators couldn’t be sure if the test user was done or just attending to another task.
	Quickly establishing rapport and relationships	At times, moderators experienced anxiety about getting technical problems solved quickly to reduce test user stress and to ensure not too much testing time was taken up by technical problems. In Project 1, the moderator was on the same campus as some of the test users and was requested to come in person to the test user’s office to set up the audio prior to the test session.
	Concept of time	The technical setup took longer than anticipated, so at times moderators felt rushed for time to complete usability tasks.
		There was no “clock” tool to help provide test users or moderators with cues about how much time a task had taken.
	Surveillance	Moderators’ virtual presence was constant and all-encompassing. Test users’ every click was monitored and every task was recorded. Moderators felt that the testing context might have led to feelings of being surveilled, obligations to have opinions, or pressure on test users to perform as expected.

While all moderators were eager to allay test user anxieties or help overcome challenges the test users might be experiencing, they also did not “want to interject too often as the goal of the session was to identify problems” [MOD 3]. Tangled conversation (ie, speaking over each other, unintentionally interrupting) was exacerbated by technical problems with audio and video play that sometimes cut-out completely or lagged, resulting in episodes of audio speeding up in order to “catch up” or audio feedback. In Project 1, testing sessions were more moderator-led, with less time for test users to explore freely and more structured interaction between the test user and moderator. Given that usability sessions had a target time limit (eg, 90 minutes) moderators needed to manage the sessions closely. It is interesting that all test users opted out of using the Simultaneous Camera feature in Blackboard Collaborate, meaning they did not see the moderator and, therefore, had no eye contact or body language to inform their communication strategies. The physical or “personal” disconnection was noted as an important factor in Project 1 more than in Project 2. Given that Project 2 had only 1 moderator and 1 test user in each session, it may be that there was less need for explicit social feedback to manage orderly conversations even if pausing frequency was sporadically difficult to gauge:

...it could be pretty quiet...just you there listening and they were working through tasks.... You’d need to check in and make sure everything was working OK if they didn’t say anything for a while.... I asked questions, then they asked questions...then I asked questions.

MOD 3

Project 2, which incorporated a largely uninterrupted, free-exploration opportunity for test users, allowed the moderator to use Blackboard Collaborate as more of a remote observation tool and less as an interactive communication tool. As Project 2 only involved test users in a single session with 1 moderator, there was less opportunity (or arguably need) to take advantage of all the advanced communication features. The moderator observed how the test user was interacting with the online intervention through a shared desktop and could answer questions verbally as or if needed. Since there was only 1 moderator and a single user in each session, the moderator found there were fewer relational dynamics to manage and fewer interaction cues to monitor (eg, who was logged in, who was speaking, who was typing in the chat box).

In Project 1, which had 2 moderators present, each moderator only had a limited amount of power to direct where the conversation went. Each moderator in that case was charged with leading a certain aspect of the test sessions, with priorities and assumptions in the mix, meaning the exchange could be pulled in any number of directions. Conversations could jump around and move away from a topic a moderator was getting ready to talk about. In the absence of visual cues, moderators in Project 1 expected that people might “talk over each other” at times and were reluctant to interject and be seen as “disrupting” or “talking over” someone else. Not infrequently, the moderators and test users interrupted each other, but for the most part there was a comfortable back and forth.

Despite only a small number of people attending usability sessions, moderators identified conversational challenges. Moderators from both projects felt remote communication mediated by the Web-conferencing tool led to more simultaneous talking and “tangled” conversation. They noted some difficulties in managing both overenthusiastic and silent test users along with offering the right level of support during think-aloud exercises. A moderator from Project 1 provided an example:

Sometimes you would talk and there was a little delay...the other person would start talking and it would get confusing...sometimes [you] needed to wait and make sure they were finished talking or else you would end up talking over and interrupting each other...and some people were really chatty and it was hard to read when to get a word in...to interject and refocus.

MOD 1

#### Theme 3: Moderators Experienced Diverse Technical Demands but Were Able to Collaboratively Troubleshoot With Test Users

Technical considerations related both to the technical infrastructure as well as the technical competency of moderators and test users. Although the overall computer competency of the test users was quite high, many had never used Web conferencing before. Given that test users were both evaluating an online eHealth intervention they were unfamiliar with and using a Web-conferencing tool, technical issues arose and developing collaborative dialogue was challenging at times for moderators. Moderators found that they were not only required to make more advanced use of the interface during the session, but also were ultimately responsible for providing live troubleshooting support. Moderators described experiencing the most significant technical difficulties around ensuring high quality audio (eg, reducing audio feedback, volume, clarity), software requirements and compatibility issues, and data export (eg, file format) for further data analysis.

You needed backup plans.... One participant was supposed to update their JavaScript before the session but didn’t.... We tried for 10 or 15 minutes and couldn’t get that fixed...[so] we ended up having them switch to a different computer altogether.... We were wasting time.

MOD 1

Moderators also expressed a certain degree of stress in “rushing” or trying to “get through” the usability protocol tasks given more time than expected had to be spent on technical issues with some test users. All moderators described instances of being affected by what they perceived as test user stress and varying degrees of technical computer competence. Moderators in Project 1 felt that they:

...didn’t really have time to learn and incorporate the ‘bells and whistles’ [of Blackboard Collaborate]...like the emoticons...[which] might have helped communication, but it would have taken time for...[test users] to learn how to use them.

MOD 2

All moderators tended to downplay the overall impact of these technical issues in terms of the quality of usability results. Technical inconveniences were primarily experienced during setup. Moderators also experienced interactions with test users that included laughing, light-hearted joking about technical prowess, and opportunities to empathize with test users around technical malfunctions.

##  Discussion

### Principal Results

Moderators’ experiences across both projects in this study have identified functional advantages and disadvantages of using Web-conferencing software for usability testing. Much has been written about how Web conferencing allows the moderator to “capitalize” on functionality that supports interaction and collaboration [29]. However, the need for moderators and test users to divide their attention among auditory, textual, and visual material makes high demands on limited working memory and may result in cognitive overload [30,31]. This kind of “disciplined improvisation” [32] presented challenges for the 2 eHealth projects we examined. Traditional usability techniques such as think-aloud and cognitive walk-through are not easily applied in dynamic, interruption-prone environments or with clinical populations who may have complex underlying motivational, cognitive, or physical challenges [33,34]. Test users in these contexts are evaluating a health technology that they are not familiar with via a usability testing mechanism that is also unfamiliar, which can create cognitively demanding test scenarios. Project 1 emphasized the need to consider cognitive load when developing usability testing methods. This would suggest that moderators engaged in lengthy usability sessions need advanced skills and knowledge to navigate the clinical, technical, online collaboration, and software development process aspects of the test sessions. Researchers should acknowledge how human factors not only affect the design and implementation of health interventions, but also the testing and vetting processes as well. The emergence of many new usability services and processes provide promising technical facilitation opportunities, but there is a need for more research evidence about how the nature of these virtual testing environments might be mediating or moderating results in unexpected ways.

### Limitations

The advanced feature set of Blackboard Collaborate (eg, Web cams, polling, emoticons) might have helped improve collaboration, but wasn’t pragmatic in short usability testing sessions like those described here, where there was limited time to learn about and develop competency in all the features. Unlike a semester-long, Web-conference-delivered course where facilitators and learners have significant time to develop proficiency and use the more advanced features of a tool, our short usability sessions required moderators and test users to quickly adapt and learn the technology. The technical and collaborative competencies of the moderator and test users may be particularly amplified for shorter usability sessions like those in our projects.

In this case study, the moderators’ challenges in creating and sustaining social presence and untangling dialogue required them to draw on their technical and interpersonal communication skills. Increased levels of interactive complexity require heightened levels of online collaborative competencies, which are supported by many online learning models [11] and may also transfer to remote synchronous usability evaluation contexts. Emerging research into self-disclosure, group norms in online communication, and use of strategies to overcome lack of nonverbal cues in computer-mediated communication [35,36,37] have potential implications for the reliability and validity of testing usability of eHealth interventions “in context” in remote online test environments. New and innovative usability platforms and services that provide access to massive pools of standardized test user data are invaluable but are not without their own limitations and bias. Researchers should not assume that any given testing strategy (ie, laboratory or remote, automated or moderated) will remove these challenges completely. This study highlights some of the human factors that shape interaction between moderators and test users in a Web-conference test environment. Given evidence in the online learning literature of the relationship between interaction and perceived effectiveness, user satisfaction, and engagement [38], it is important to better understand how interaction patterns observed in moderated remote testing might affect interpretation of usability results and ultimately influence design decisions made as a result. A more rigorous research program in the field of moderating usability testing for eHealth interventions could lead to improved training, the development of better testing tools/platforms, and more refined usability measures.

Findings suggest that moderators of usability sessions face diverse technical demands but are, if experienced with the technology, able to collaboratively troubleshoot with test users. Research suggests that some of these interaction challenges create additional stress for moderators. Moderators might infer that test users need assistance, but it can be difficult to know when to interject or offer support [39] without confounding usability test results. While some would suggest that a nonexpert moderator’s failure to understand subtle features of the tool or its use might have a crippling impact on the usability session, we found that, generally, test users and moderators demonstrated considerable technical and collaborative competencies to preempt technical challenges or troubleshoot and resolve issues together during the sessions. Perhaps ironically, technical challenges seemed to create opportunities to show empathy and humanize the moderator-tester relationship—particularly in the first few minutes of the test session when rapport was just being established.

### Conclusions

If current trends continue, the general population will become increasingly familiar with Web-conferencing tools through formal education [40]. Moderators will increasingly require online collaborative skills to navigate test user needs, resolve technical challenges, and accommodate “real life” events that may unexpectedly appear during the testing process. As competencies with Web conferencing increase, many of the issues highlighted in this paper might be overcome and the benefits of remote testing more easily realized.

It may be helpful to formally investigate the relationships between moderators’ experiences and their personal characteristics (including previous usability experience and private theories about “good design”) to help researchers understand how to best prepare moderators to support test users in virtual environments. While laboratory settings allow for more experimental control and collection of various types of data, these settings lack the realism of a field setting and deeper engagement from potential end users into hedonic quality factors that impact satisfaction [41]. “How-to” books and resources for facilitating and moderating usability sessions and training programs for usability testers are becoming more accessible, but published peer-reviewed research on moderator experiences is lacking. Insights from moderator-focused studies might be advantageous in designing test environments that put less cognitive burden on all test scenario test users. Comparative research is also needed to better understand how cognitive load and technical competence might moderate results in remote versus in-person usability testing [42]. Understanding a moderator’s role in usability testing, as well as the influences and impacts their role can have, requires a sociotechnical framework that accounts for the complex interactions between human behavior and actions and the tools and technologies in the environment [43].

## Abbreviations

CIHR: Canadian Institutes of Health Research
FASD: fetal alcohol spectrum disorder
IRIS: Intelligent Research and Intervention Software
